# Comprehensive transcriptome analysis reveals MSTRG.19853.1/ssc-miR-361-3p/*NPPA* axis is related to hypoxic adaptation in Tibetan pigs

**DOI:** 10.1186/s12864-025-11783-8

**Published:** 2025-07-01

**Authors:** Pan Li, Wei Cheng, Zhandui Pubu, Peng Shang, Hao Zhang, Bo Zhang

**Affiliations:** 1 Frontiers Science Center for Molecular Design Breeding (MOE), China Agricultural University, Beijing, 100193 China; 2 National Engineering Laboratory for Animal Breeding, Beijing Key Laboratory for Animal Genetic Improvement, College of Animal Science and Technology, China Agricultural University, Beijing, 100193 China; 3 Institute of Animal Husbandry and Veterinary, Xizang Academy of Agricultural and Animal Husbandry Sciences, Lhasa, 85000 China; 4 Department of Animal Husbandry, Xizang Agricultural and Animal Husbandry University, Linzhi, 860000 China

**Keywords:** Tibetan pig, Hypoxic adaptation, ceRNA network, Non-coding RNAs

## Abstract

**Background:**

The Tibetan pig, an indigenous breed adapted to plateau environments in China, exhibits remarkable tolerance to extreme high-altitude conditions. Recent studies have highlighted the pivotal role of non-coding RNAs (ncRNAs) in regulating hypoxic adaptation. However, the complex regulatory network involving mRNAs and ncRNAs that mediate this adaptation in Tibetan pigs remains poorly understood.

**Results:**

We performed whole-transcriptome sequencing to analyze expression profiles of mRNAs, lncRNAs, and miRNAs in heart tissues of Tibetan pigs (TH) and Yorkshire pigs (YH) at high altitude. We identified 795 differentially expressed lncRNAs (DE lncRNAs), 149 differentially expressed miRNAs (DE miRNAs), and 2,206 differentially expressed mRNAs (DE mRNAs) between TH and YH. Functional enrichment analysis showed that target genes of DE miRNAs, DE lncRNAs, and DE mRNAs significantly enriched pathways related to hypoxic adaptation, including Dilated Cardiomyopathy (DCM) and Hypertrophic Cardiomyopathy (HCM). We constructed a competing endogenous RNA (ceRNA) regulatory network comprising 8 DE lncRNAs, 37 DE miRNAs, and 7 DE mRNAs. Notably, we validated the MSTRG.19853.1/ssc-miR-361-3p/*NPPA* axis, a candidate regulator of cardiac adaptation, using quantitative real-time PCR (qRT-PCR) and dual-luciferase reporter assays.

**Conclusion:**

Our findings elucidate comprehensive RNA expression profiles and ncRNA-mRNA interactions underlying hypoxic adaptation in Tibetan pig hearts compared to Yorkshire pigs at high altitude. The MSTRG.19853.1/ssc-miR-361-3p/*NPPA* axis represents a promising candidate for regulating cardiac adaptation under hypoxia, pending in vivo validation. These insights enhance our understanding of the genetic mechanisms driving high-altitude adaptation in Tibetan pigs, offering a foundation for comparative studies of hypoxic resilience in plateau mammals.

**Graphical Abstract:**

The model of MSTRG.19853.1/ssc-miR-361-3p/*NPPA* axis for regulating hypoxia adaptation in Tibetan and Yorkshire pigs.

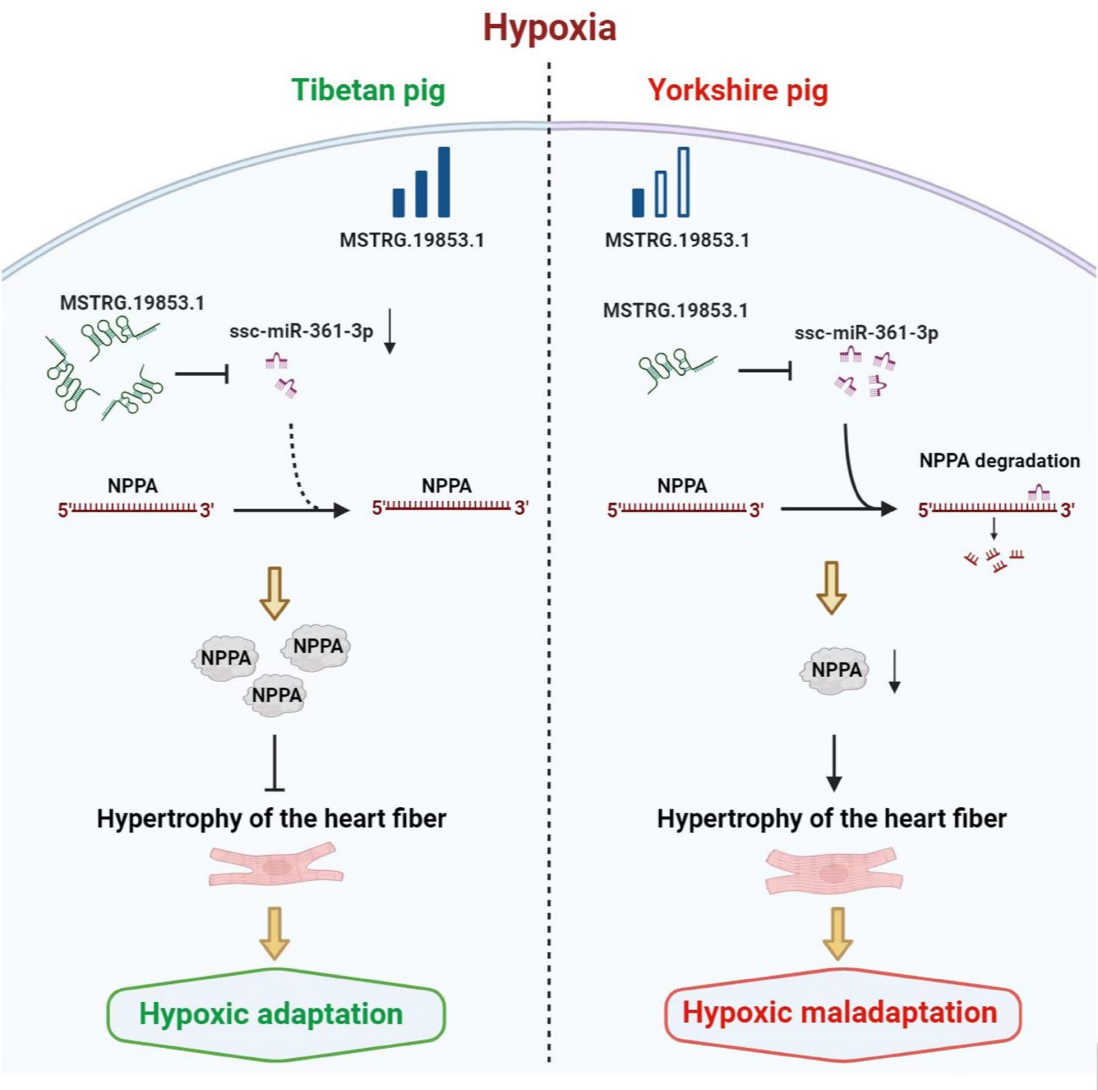

**Supplementary Information:**

The online version contains supplementary material available at 10.1186/s12864-025-11783-8.

## Background

Surviving in the hypoxic environment of high-altitude plateaus poses significant physiological challenges for low-altitude mammals. The unique geography of these regions has been leveraged to study the effects of hypoxia on mammalian physiology, with the aim of improving our understanding and treatment of hypoxia-related conditions in biological and medical contexts [1, 2]. The Tibetan pig (*Sus scrofa*), an indigenous breed distributed across the Qinghai-Tibet Plateau at altitudes exceeding 2500 m, has evolved heritable genetic adaptations in anatomical, physiological, biochemical, and other traits through evolutionary processes. These adaptations make it easier for Tibetan pigs to survive under low oxygen tension compared to low-altitude pigs [3, 4]. High-altitude mammals, including Tibetan pigs, exhibit systemic adaptations in cardiovascular, respiratory, and hematopoietic functions to enhance convective oxygen transport [5]. For instance, Tibetan pigs possess arteries with smaller inner diameters, thicker vessel walls, and higher blood physiological and biochemical indices compared to low-altitude pigs [6, 7]. These adaptations make the Tibetan pig an ideal model for studying the molecular mechanisms of hypoxia. Despite advances in understanding DNA methylation [8], proteomics [9], mRNA [9], and miRNA [10] expression profiles in Tibetan pig hearts, the comprehensive transcriptome and the interplay between mRNA and non-coding RNAs (ncRNAs) remain poorly characterized. Furthermore, studying hypoxic adaptation on plateaus requires the integrated regulation and synergistic interaction of multiple genetic and molecular levels [11].

The advent of high-throughput sequencing has revolutionized our understanding of ncRNAs, which were once considered ‘junk DNA,’ by revealing their crucial roles in cellular physiology and pathology [12]. ncRNAs are categorized into lncRNAs, miRNAs, and circRNAs based on size and structure [13]. lncRNAs and miRNAs, as linear ncRNAs, are implicated in a variety of biological processes, including the hypoxic response [14, 15], metabolic regulation of lipids and glucose [16], and cancer development [17]. The regulatory mechanisms of lncRNAs and miRNAs differ, with each playing distinct roles in cellular function, suggesting their involvement in hypoxia-related gene regulation. For instance, the cooperative interaction between lncRNA and Ezh2 suppresses HIF-1α transcription, facilitating cancer cell adaptation to hypoxia [18]. Evidence is mounting that lncRNAs regulated by hypoxia are involved in cancer cell progression and HIF-1α-mediated transcriptional activation. In terms of high-altitude hypoxic acclimation, a study on yaks has identified some potential lncRNAs using transcriptomic approaches and suggested their potential regulatory functions in yaks for high-altitude hypoxic acclimation [19]. Similarly, lncRNAs associated with hypoxic adaptation in Tibetan chickens have been identified, highlighting their roles in oxygen transport and signaling [20]. However, knowledge of ncRNAs in Tibetan pigs is scarce. The competing endogenous RNAs (ceRNAs) hypothesis proposes that lncRNAs can sponge miRNAs, alleviating their suppressive effects on target genes and modulating post-transcriptional gene expression. A potential lncRNA-miRNA-mRNA regulatory pathway, based on shared miRNAs, has been proposed. Emerging evidence highlights the critical role of the ceRNA network in hypoxic adaptation and heart development [21].

The heart, as the dominant organ of the circulatory system, is pivotal in animal adaptation and response to hypoxia, enhancing our comprehension of the physiological and pathological changes that occur under such conditions [22, 23]. High-altitude adaptations, such as increased heart rate and blood pressure, can induce structural and functional cardiac alterations. Notably, Tibetan pigs exhibit enhanced cardiac function and superior hypoxic tolerance compared to low-altitude pigs [3, 8]. This study assessed the physiological cardiac phenotypes of Tibetan and Yorkshire pigs to uncover the cardiac adaptive mechanisms of Tibetan pigs to the extreme hypoxic plateau environment. Additionally, we conducted a comprehensive analysis of lncRNA and miRNA transcripts and their features in the heart tissues of Tibetan pigs (TH) and Yorkshire pigs (YH), constructing a ceRNA network encompassing lncRNAs, miRNAs, and mRNAs. This network aims to identify epigenetic factors associated with hypoxic adaptation in Tibetan pigs. Our findings contribute to elucidating the intricate regulatory mechanisms at play and offer novel insights into the hypoxic adaptation processes of Tibetan pigs.

## Materials and methods

### Ethics statement

All animal experiment care protocols were approved by the Animal Welfare Committee of the State Key Laboratory for Agro-biotechnology of the China Agricultural University (Approval number XK257), and all experiments were performed in compliance with approved relevant guidelines and regulations.

### Experimental animals and sample collection

The experimental animals comprised two groups: Tibetan pigs and Yorkshire pigs, both reared at a high-altitude (Linzhi, Tibet, China; Altitude: 3000 m above mean sea level; Geographic coordinates: 29°38’N, 94°48’E). Tibetan pigs living at high-altitude were designated as TH, while Yorkshire pigs living at high-altitude were designated as YH. Two groups (total *n* = 6) were selected from healthy six-month-old pigs of similar weights, all meeting standard slaughter readiness criteria. Following humane euthanasia via electrical stunning and exsanguination, heart tissues were promptly excised for analysis. After removing the atria and excess tissue, the right ventricle was sectioned along the septum and dried using filter paper. The whole ventricle (WV) and right ventricle (RV) were weighed using an electronic scale. Subsequently, the right ventricular hypertrophy index (RVHI) was calculated using the formula: RVHI = (RV/WV) × 100% serving as an indicator of right ventricular hypertrophy [24]. Physiological phenotypic changes in heart tissue were evaluated through hematoxylin and eosin (H&E) staining. Additionally, heart tissue samples were collected, frozen in liquid nitrogen, and stored at − 80 °C for total RNA extraction.

### Heart histology

Heart tissues were immobilized in 4% paraformaldehyde for 30 min and embedded in paraffin to prepare paraffin sections. The collected heart tissues were transected to obtain cross-sections. The sections were stained with hematoxylin for 15 min, differentiated in 1% hydrochloric acid-ethanol for a few seconds, and then stained with 0.5% eosin for 3 min. This was followed by gradient alcohol dehydration for 2 min and vitrification with dimethylbenzene for 2 min. The cross-sections were observed and photographed under a microscope (using an Echo Revolve, Discover Echo Inc., San Diego, CA, USA).

### RNA extraction and quality control

Total RNA was extracted from six heart tissue samples (Group TH: TH1, TH2, TH3; Group YH: YH1, YH2, YH3) using TRIzol^®^ Reagent (Ambion Inc., Austin, TX, USA) according to the manufacturer’s instructions. The quality and purity of the total RNA samples were assessed using 1% agarose gel electrophoresis and a NanoDrop™ 2000 Spectrophotometer (Thermo Fisher Scientific Inc., Wilmington, DE, USA). RNA integrity was evaluated with the Agilent Bioanalyzer 2100 system (Agilent Technologies Inc., Santa Clara, CA, USA). All six samples had an RNA integrity number (RIN) ≥ 7 and were subjected to subsequent sequencing analyses.

### High-throughput sequencing of RNA library construction and sequencing

A total of 3 µg of each RNA sample was utilized for library preparation. For mRNA and lncRNA sequencing, total RNA was treated with the Epicentre Ribo-ZeroTM rRNA Removal Kit (Epicentre Technologies Corp, Madison, WI, USA) to remove ribosomal RNA (rRNA). Libraries were constructed using the NEBNext^®^ Ultra™ Directional RNA Library Prep Kit for Illumina^®^ (New England Biolabs, Ipswich, MA, USA) following the manufacturer’s instructions. Briefly, RNA was fragmented, and the first-strand cDNA was synthesized using random hexamers, followed by second-strand cDNA synthesis with dNTPs, RNase H, and DNA Polymerase I. After purification with the QiaQuick PCR kit and elution with EB buffer, the fragments underwent end repair, poly-adenylation, adapter ligation, and agarose gel electrophoresis before PCR amplification. Constructed libraries were assessed using the Agilent Bioanalyzer 2100 system and sequenced on an Illumina Novaseq 6000 platform with 150 bp paired-end reads. For miRNA sequencing, small RNA libraries were constructed using the NEBNext^®^ Multiplex Small RNA Library Prep Set for Illumina^®^ (New England Biolabs, Ipswich, MA, USA) with 3 µg of RNA per sample, following the manufacturer’s instructions. Briefly, the 3’ SR adaptor was ligated to the 3’ end of small RNAs using T4 RNA ligase. The SR reverse transcription primer was hybridized to the 3’ SR adaptor to form a double-stranded structure, followed by ligation of the 5’ SR adaptor. The adaptor-ligated small RNAs were reverse-transcribed into cDNA, amplified by PCR, and size-selected (~ 140 bp, corresponding to miRNAs) using 6% polyacrylamide gel electrophoresis. Library quality was assessed using the Agilent Bioanalyzer 2100 system. The libraries were sequenced on an Illumina Novaseq 6000 platform with 50 bp single-end reads.

### Identification of LncRNAs and MiRNAs

Raw reads from each sample were filtered to obtain clean reads by removing adapters, reads containing more than 10% unknown nucleotides (N), and low-quality reads. Clean reads were mapped to the pig reference genome (http://ftp.ensembl.org/pub/release103-/fasta/sus_scrofa/dna/Sus_scrofa.Sscrofa11.1.dna.toplevel.fa.gz) using HISAT2 [25]. StringTie [26] assembled the reads for each sample, and GffCompare [27] annotated the assembled transcripts. Unknown transcripts were screened to identify putative lncRNAs. Candidate lncRNAs were selected based on the following criteria: (1) Only transcripts with class codes “i”, “x”, “u”, “o”, and “e” representing potential novel intergenic, intronic, and cis-antisense transcripts were selected [28]. (2) Transcripts with a length of ≥ 200 bp and an exon number of ≥ 2 were selected [29]. (3) Transcripts with an FPKM value ≥ 0.1 were selected [29]. (4) Finally, the coding potential of the candidate lncRNAs was predicted using Coding Potential Calculator (CPC2) [30], Coding-Non-Coding Index (CNCI) [31], Coding Potential Assessment Tool (CPAT) [32] and Pfam [33]. (5) Different types of lncRNAs, including lincRNA, intronic lncRNA, antisense lncRNA, and sense lncRNA, were identified using CuffCompare. For miRNA data, clean reads were obtained using custom Perl scripts and aligned with small RNAs from the Silva, GtRNAdb, Rfam, and Repbase databases to identify and remove rRNA, transfer RNA (tRNA), small nucleolar RNA (snoRNA), and small nuclear RNA (snRNA). All unannotated reads were aligned to the pig reference genome using Bowtie software [34]. Known miRNAs were identified by aligning mature miRNA sequences to miRBase (v22) using miRDeep2 [35]. Novel miRNAs were predicted using miRDeep2 (v2.0.1.2) with the following criteria: a minimum score threshold of 0, precursor length of 60–100 nt, Randfold *P* < 0.05, and minimum free energy (MFE) ≤ −20 kcal/mol to ensure reliable identification of miRNA precursors with stable hairpin structures. The pipeline automatically assigned temporary identifiers (novel-miR-[number]) through sequential numbering of predicted candidates, with the ‘novel-’ prefix clearly distinguishing these from known miRBase (v22) annotations.

### Analysis of DE mRNA and DE NcRNA

To analyze the expression of mRNAs and lncRNAs, clean reads were aligned to the pig reference genome (Sscrofa11.1) using HiSAT2 [25] to generate mapped reads. These reads were then assembled into transcripts using StringTie [26], which also quantified gene expression normalized by Fragments per kilobase of exon model per million mapped fragments (FPKM). Differential expression analysis was performed using the DESeq2 package [36]. Differentially expressed lncRNAs (DE lncRNAs) and differentially expressed mRNAs (DE mRNAs) were identified based on an FDR < 0.05 and|log₂FoldChange| ≥ 2. For miRNA expression, levels were quantified using Transcripts Per Kilobase of exon model per Million mapped reads (TPM). Differentially expressed miRNAs (DE miRNAs) were identified based on|log₂FoldChange| ≥ 1 and *P* < 0.05. The lower fold-change threshold (|log_2_FoldChange| ≥ 1) for miRNAs was chosen due to their typically smaller expression changes compared to mRNAs and lncRNAs, while *P* < 0.05 was used given the smaller number of miRNAs detected (typically hundreds compared to tens of thousands for mRNAs/lncRNAs), which reduces the multiple testing burden. This approach, combined with subsequent quantitative real-time PCR (qRT-PCR) validations, ensured reliable identification of biologically significant miRNAs [37].

### Prediction of NcRNA target genes and enrichment analysis

LncRNAs regulate target genes through cis- or trans-regulation, forming lncRNA-mRNA pairs. Therefore, two regulatory modes are integrated to predict the target genes of lncRNAs: (1) Prediction of lncRNA regulation on adjacent coding genes based on their positional relationship, where adjacent coding genes within 100 kb of the lncRNA are considered as its target genes; (2) Prediction based on significant positive correlations between lncRNA expression and potential target gene expression across all samples utilizing correlation analysis to predict trans-target genes of lncRNAs. DE lncRNA/DE mRNA pairs were screened using Pearson correlation analysis with a threshold of|r| ≥ 0.9 and *P* < 0.05. DE mRNAs were further filtered based on KEGG pathway annotations (*P* < 0.05) relevant to hypoxic adaptation. Finally, a DE lncRNA/DE mRNA network was constructed and visualized using Cytoscape 3.6.1 software [38]. Target genes of DE miRNAs were predicted using miRanda [39] and TargetScan [40] based on miRNA and gene sequence information from the pig reference genome (Sus scrofa, Sscrofa11.1). To explore the biological functions of DE mRNAs, DE lncRNA target genes, and DE miRNA target genes, KEGG pathway enrichment analyses were performed using the KOBAS (v3.0) [41, 42]. KEGG pathways with *P* < 0.05 were considered significantly enriched.

### CeRNA regulatory network construction

The interaction pairs for miRNA-mRNA and miRNA-lncRNA were predicted using miRanda and TargetScan. Based on the expression levels of DE mRNAs, DE miRNAs, and DE lncRNAs, Pearson’s correlation coefficients and *P*-value were calculated for both miRNA-mRNA and miRNA-lncRNA pairs. Negatively correlated pairs with a *P* < 0.05 were selected for further analysis. Shared pairs which were predicted from binding sites by software, and the predicted pairs from the expression levels of DE mRNAs, DE miRNAs, and DE lncRNAs were utilized for the next step. Finally, we constructed a lncRNA-miRNA-mRNA network of interest by integrating the three relationship pairs (DE lncRNAs/DE mRNAs, DE miRNAs/DE mRNAs, and DE miRNAs/DE lncRNAs) and visualized it using Cytoscape 3.6.1 software.

### Verification of qRT-PCR

To confirm the accuracy of RNA-seq expression levels, six lncRNAs, six miRNAs, and six mRNAs (listed in Table S1) were selected for qRT-PCR validation based on their significant differential expression (|log_2_FoldChange| ≥ 2 for lncRNAs/mRNAs, ≥ 1 for miRNAs), functional relevance to hypoxic adaptation. For qRT-PCR of mRNAs and lncRNAs, cDNA was synthesized using the FastKing RT Kit (TIANGEN Biotech (Beijing) Co., Ltd., Beijing, China). qRT-PCR was performed using the SYBR Green kit (TIANGEN Biotech (Beijing) Co., Ltd.) on a Bio-Rad CFX96 Real-Time PCR System (Bio-Rad Laboratories, Inc., Hercules, CA, USA) according to the manufacturer’s instructions. For qRT-PCR of miRNAs, the miRcute Plus miRNA First-Strand cDNA Kit (TIANGEN Biotech (Beijing) Co., Ltd.) was used to synthesize first-strand cDNA for miRNA expression analysis. qPCR was performed using a miRcute Plus miRNA qPCR Kit (SYBR Green) (TIANGEN Biotech (Beijing) Co., Ltd.) with the provided miRNA reference gene (U6) on the same Bio-Rad CFX96 Real-Time PCR System. The 2^–ΔΔCT^ method was used to quantify the relative gene expression levels. qRT-PCR primers for lncRNA and mRNA were designed using NCBI Primer-BLAST (National Center for Biotechnology Information, Bethesda, MD, USA) [43], and qRT-PCR forward primers for miRNA were designed using miRprimer2 [44]. All primers were synthesized by BeiJing Genomics Institute (BGI, Beijing, China) (Table S1).

### Dual-Luciferase reporter assay

The interaction between MSTRG.19853.1 and ssc-miR-361-3p, and between ssc-miR-361-3p and *NPPA*, was validated using a dual-luciferase reporter assay in chicken Dermal Fibroblast 1 (DF1) cells (ATCC, CRL-12203) due to their high transfection efficiency. The oligonucleotides used in this study, including miR-361-3p mimic and mimic negative control (NC), were synthesized by Gene Pharma Co., Ltd. (Shanghai, China) and are listed in Table S1. Wild-type (WT) and mutant (Mut) sequences of MSTRG.19853.1 and *NPPA* 3’ UTR containing miR-361-3p binding sites were synthesized and cloned into the psi-CHECK^TM^−2 vector by Tsingke Biotechnology Co. Ltd (Beijing, China). The transfection treatment combinations used in the dual-luciferase reporter assay were “WT + miR-361-3p mimic,” “Mut + miR-361-3p mimic,” “WT + mimic NC” and “Mut + mimic NC”. DF 1 cells were plated in 24-well plates, and co-transfected in triplicate with 80 nmol/L of miR-361-3p mimics or mimics NC and 500 ng of the aforementioned WT or Mut plasmids per well. The dual-luciferase reporter assay was performed using a dual-luciferase reporter assay kit (Nanjing Vazyme Biotech Co. Ltd., Nanjing, China), according to the manufacturer’s protocol. Firefly luciferase and Renilla luminescence activities were measured using a multifunction microplate reader (BioTek Instruments, Inc., Winooski, VT, USA).

### Data analysis

All statistical analyses were performed using SPSS version 23.0 software. Data were represented as the mean ± standard error, and statistical significance was assessed using an unpaired Student’s t-test for dual-luciferase reporter assay analysis, whereas one-way ANOVA with the Student-Newman-Keuls post hoc test was applied for RVHI and qRT-PCR analysis. A *P* < 0.05 was considered indicative of significant differences.

## Results

### Physiological phenotypic characteristics of heart tissue in Tibetan pig (TH) compared to Yorkshire pig (YH)

**Fig. 1:**
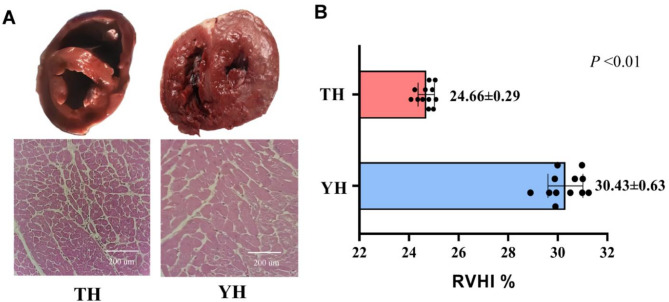
Physiological phenotype data of the heart. **A** H&E staining of heart tissues in the Tibetan pig (TH) and Yorkshire pig (YH) at high-altitude. **B** Comparison of the right ventricular hypertrophy index (RVHI) between the TH and YH. Data are represented as the mean ± (standard error) SE

To explore the physiological phenotypic differences in heart tissue between TH and YH, we examined the RVHI and the diameter of individual myofibers. H&E staining revealed that the diameter of individual myofibers in YH was larger than in TH (Fig. 1A). The RVHI of YH was 30.43%, significantly higher than that of TH (*P* < 0.05) (Fig. 1B). These findings suggest that YH exhibits a maladaptive phenotype, characterized by abnormal hypertrophy of individual myofibers and elevated RVHI levels.

### Overview of RNA transcriptome profiles in pig heart tissue

**Fig. 2:**
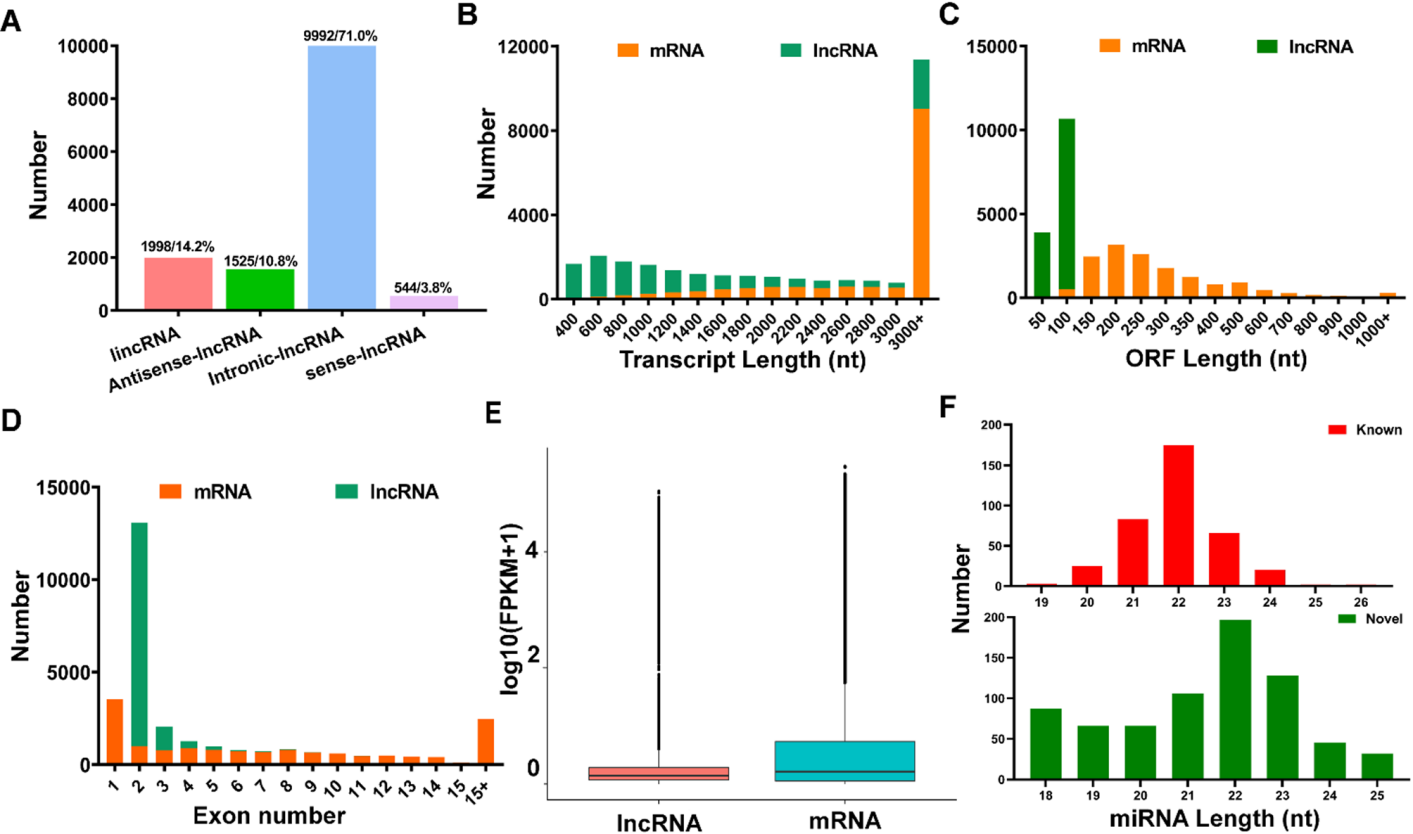
Comparison of the features of RNAs in the pig hearts. **A** Proportions of lncRNAs subclasses, including lincRNAs, antisense lncRNAs, intronic lncRNAs, and sense lncRNAs. **B** Distribution of transcript lengths for mRNAs and lncRNAs. **C** Distribution of open reading frame (ORF) lengths for mRNAs and lncRNAs. **D** Distribution of exon numbers for mRNAs and lncRNAs. **E** Expression level indicated by log_10_ (FPKM + 1) analysis for lncRNAs and mRNAs. **F** Distribution of lengths for known and novel miRNA reads

To investigate the molecular basis of these phenotypic differences, we conducted strand-specific RNA sequencing of heart tissues from TH and YH. Strand-specific library sequencing produced 101.46 Gb of clean bases from six heart tissue samples, with 93.71–94.21% mapping to the pig reference genome (Sscrofa11.1). The miRNA library sequencing generated 144.39 million clean reads, with Q30 bases ranging from 94.92 to 97.29% (Table S2). A total of 14,786 mRNAs, 14,059 lncRNAs, and 1,093 miRNAs (366 known and 727 novel) were identified in the heart tissues of Tibetan and Yorkshire pigs. The lncRNAs were predominantly intronic-lncRNAs (71.0%), followed by lincRNAs (14.2%), antisense lncRNA (10.8%), and sense lncRNA (3.8%) (Fig. 2A). The mRNAs primarily comprised protein-coding transcripts, with 61.13% (9,038/14,786) exceeding 3,000 nt in length, compared to only 16.62% (2,336/14,059) of lncRNAs (Fig. 2B). Compared to mRNAs, lncRNAs exhibited shorter open reading frame (ORF) lengths and fewer exon (Fig. 2C, D), and their global expression levels were lower, as shown by log_10_(FPKM + 1) analysis (Fig. 2E). For miRNAs, the majority of miRNA reads were 20 to 24 nt in length, with the most prevalent length being 22 nt, aligning with the typical output of Dicer processing and confirming their authenticity (Fig. 2F).

### Differential expression analysis of RNAs between TH and YH

**Fig. 3:**
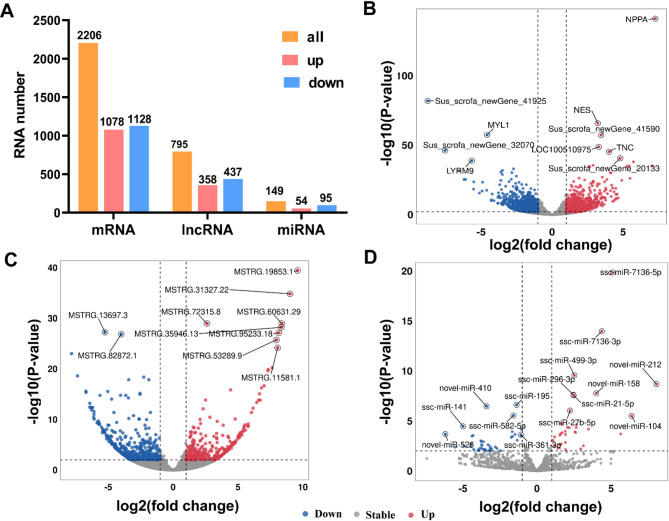
Identification of differentially expressed (DE) mRNA, DE lncRNAs, and DE miRNAs between Tibetan (TH) and Yorkshire pig (YH) at high-altitude. **A** Histogram of the number of DE mRNAs, DE lncRNAs, and DE miRNA. **B** Volcano plot of DE mRNAs. **C** Volcano plot of DE lncRNAs. **D** Volcano plot of DE miRNAs

To identify potential candidate genes related to hypoxic adaptation, the expression levels of mRNAs, lncRNAs, and miRNAs were examined in heart tissues from Tibetan and Yorkshire pigs. By comparing TH vs. YH, a total of 2,206 DE mRNA (1,078 upregulated and 1,128 downregulated; FDR < 0.05,|log_2_FoldChange| ≥ 2), 795 DE lncRNAs (358 upregulated and 437 downregulated; FDR < 0.05,|log_2_FoldChange| ≥ 2), and 149 miRNAs (54 upregulated and 95 downregulated; *P* < 0.05,|log_2_FoldChange| ≥ 1) were obtained (Fig. 3A, Table S3). As depicted in volcano plot, *NPPA* was the most upregulated gene with a 52.81-fold change, while *Sus_scrofa_newGene_41925* exhibited the highest downregulation with a 77.00-fold change in the comparisons between TH and YH (Fig. 3B). Additionally, MSTRG.19853.1 and novel-miR-212 showed the greatest upregulation with 91.98-fold and 64.99-fold changes, respectively (Fig. 3C, D). Conversely, MSTRG.29512.12 and novel-miR-526 displayed the most significant downregulation with 61.34-fold and 38.29-fold changes, respectively.

### Functional enrichment analysis of DE LncRNAs and DE MiRNAs

**Fig. 4:**
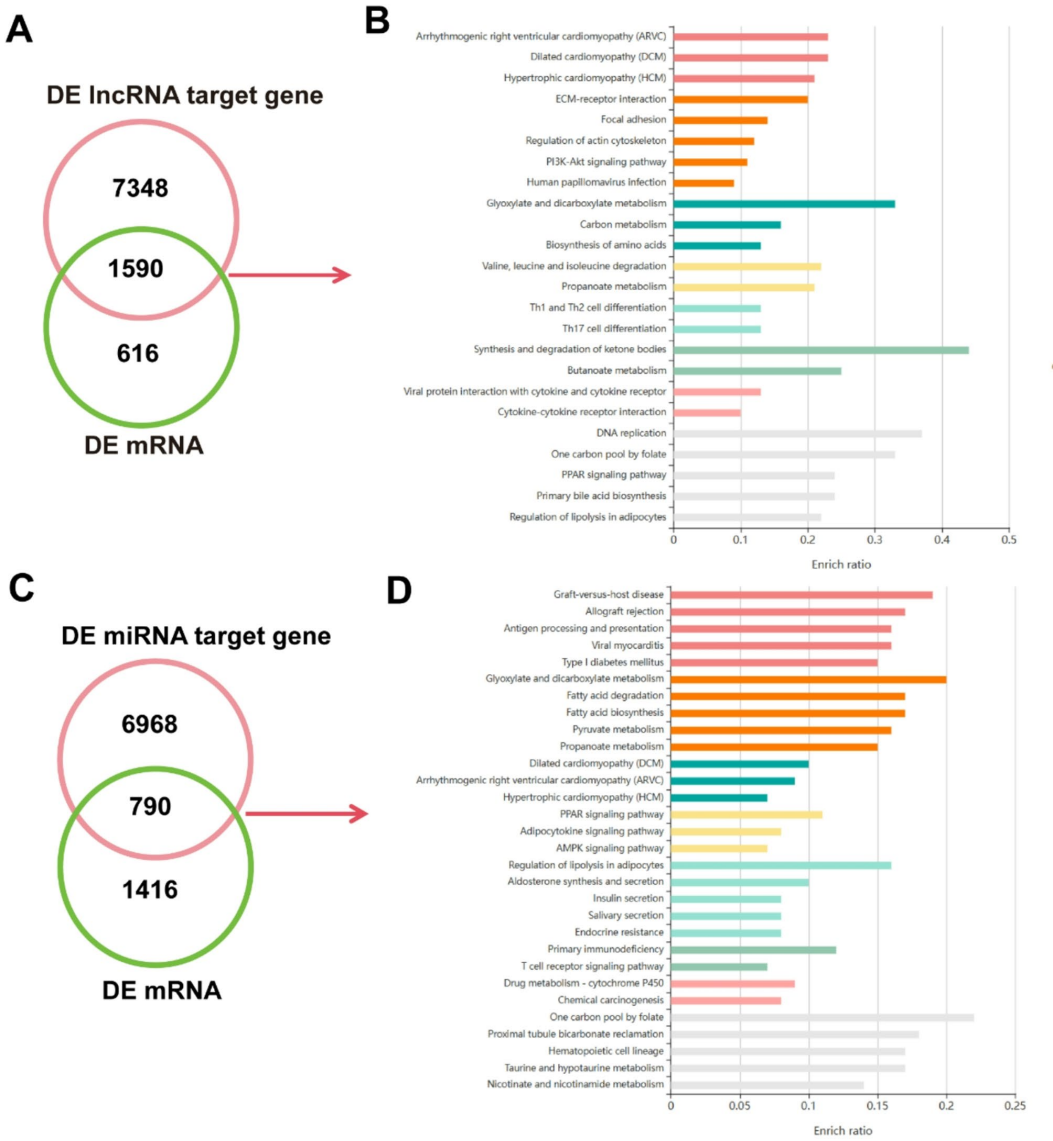
Functional enrichment analysis of differentially expressed (DE) lncRNA and DE miRNA target genes. **A** Venn diagram of the intersection between the DE lncRNA target genes and DE mRNAs. **B** KEGG enrichment analysis of 1,590 DE target genes of DE lncRNAs. Bars represent enriched pathways (*P* < 0.05), with lengths showing the enrichment ratio (Input number/Background number). **C** Venn diagram of the intersection between the DE miRNA target genes and DE mRNAs. **D** KEGG enrichment analysis of 790 DE target genes of DE miRNAs. Bars depict enriched pathways (*P* < 0.05), with lengths indicating the enrichment ratio (Input number/Background number)

To detect a reliable regulatory profile of hypoxic adaptation-related lncRNAs and miRNAs in Tibetan pig protein-coding genes, we conducted target gene prediction. This analysis yielded 94,330 lncRNA–mRNA interaction pairs involving 795 DE lncRNAs, and 8,938 target genes were identified in TH vs. YH. Among these predicted target genes, 1,590 overlapped with DE mRNAs (Fig. 4A). Functional enrichment analysis of these 1,590 DE target genes revealed 114 significantly enriched pathways (*P* < 0.05) (Table S4). Prominent pathways included cardiovascular-related ones such as Arrhythmogenic Right Ventricular Cardiomyopathy (ARVC) (enrich ratio: 0.2267), Dilated Cardiomyopathy (DCM) (enrich ratio: 0.2258), and Hypertrophic Cardiomyopathy (HCM) (enrich ratio: 0.2135), involving genes like *NPPA*, *ITGB1*, and *CACNA2D2*. Metabolic pathways, including Glyoxylate and Dicarboxylate Metabolism (enrich ratio: 0.3333) and Synthesis and Degradation of Ketone Bodies (enrich ratio: 0.4444), featured *ACAT2* and *ENO1*, indicating metabolic reprogramming for hypoxic adaptation. Importantly, The HIF-1 Signaling Pathway (enrich ratio: 0.0648) was also enriched, involving *NPPA*, *ENO1*, and *LDHA*, highlighting lncRNA-mediated regulation of hypoxia response (Fig. 4B). For DE miRNAs, 53,742 miRNA-mRNA pairs were identified, comprising 145 DE miRNAs and 7,758 target genes, of which 790 were DE (Fig. 4C). KEGG analysis of these 790 genes identified 89 significantly enriched pathways (*P* < 0.05) (Table S4). Immune-related pathways like Hematopoietic Cell Lineage (enrich ratio: 0.1705) and Th1 and Th2 Cell Differentiation (enrich ratio: 0.1319) were prominent, involving *CD38*, *IL2RA*, and *STAT4*. Metabolic pathways such as Metabolic Pathways (enrich ratio: 0.0519) and Fatty Acid Degradation (enrich ratio: 0.1707) included *ACAT2* and *ALDH2*. The HIF-1 Signaling Pathway (enrich ratio: 0.0648) was also enriched, featuring *NPPA* and *ENO1*, consistent with lncRNA results. Additionally, DCM (enrich ratio: 0.0968) involved *NPPA*, *CACNA2D2*, and *ITGB1*, reinforcing the cardiovascular focus (Fig. 4D).

### Co-expression interaction network construction

**Fig. 5:**
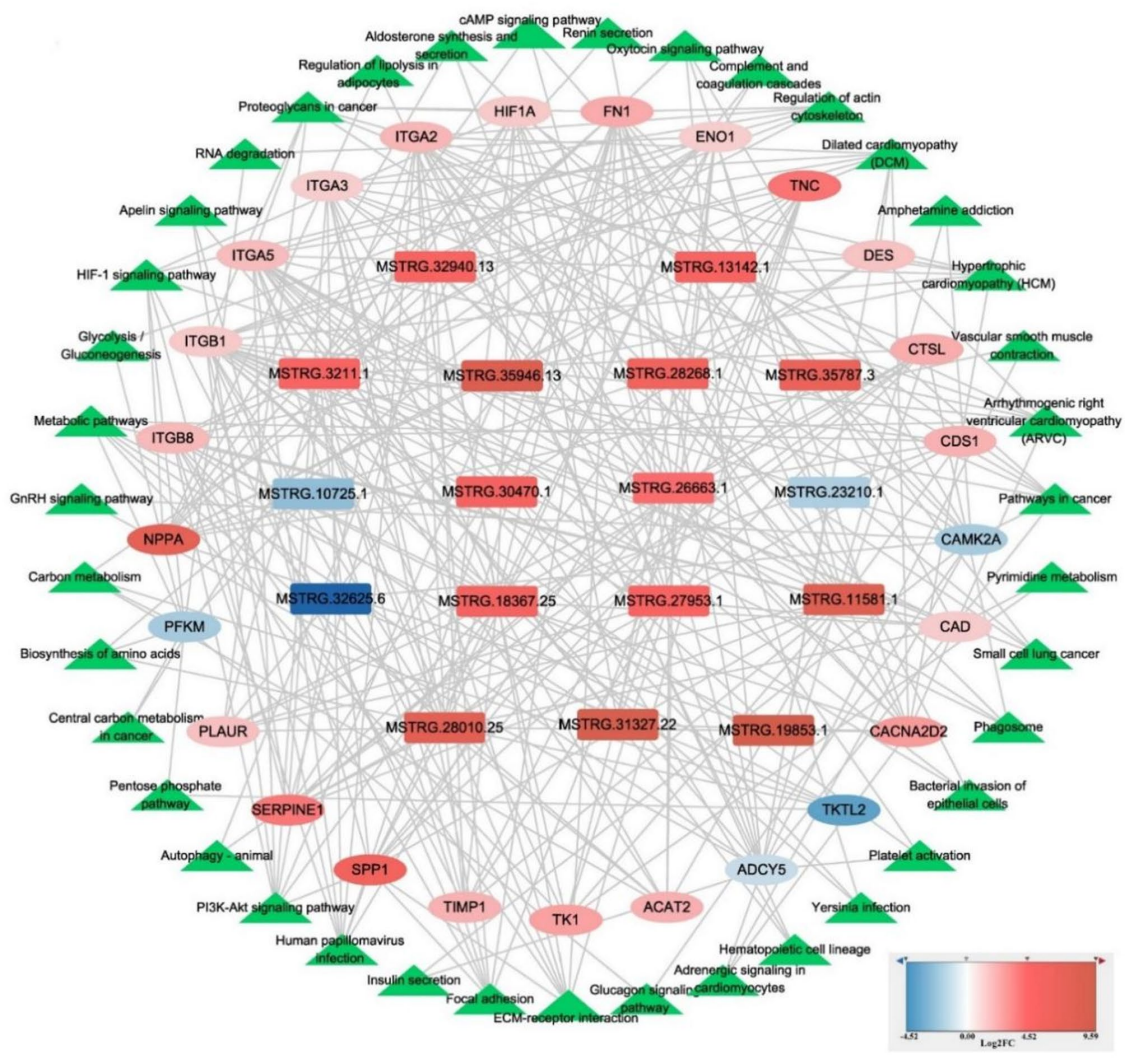
Construction of the differentially expressed (DE) lncRNA/DE mRNA interaction network construction in Tibetan (TH) and Yorkshire (YH) pigs at high-altitude. The round rectangle, ellipse, and triangles indicate DE lncRNAs, DE mRNAs, and Kyoto Encyclopedia of Genes and Genomes (KEGG) signaling pathways, respectively

To investigate the regulatory roles of DE lncRNAs, we constructed a co-expression interaction network of DE lncRNAs and DE mRNAs. We screened for DE lncRNA/DE mRNA pairs with positive co-expression and filtered key DE mRNAs associated with hypoxic adaptation based on literature and KEGG pathway analysis. The resulting network included 133 DE lncRNA/DE mRNA pairs, comprising 17 DE lncRNAs and 25 DE mRNAs (Fig. 5, Table S5). Among these, MSTRG.19853.1 (log_2_FoldChange = 9.5908), MSTRG.31327.22 (log_2_FoldChange = 9.5908), MSTRG.11581.1 (log_2_FoldChange = 8.0530), MSTRG.28010.25 (log_2_FoldChange = 7.6290), and MSTRG.35787.3 (log_2_FoldChange = 6.4539) were the top five upregulated DE lncRNAs. Notably, MSTRG.19853.1 showed high positive correlations with *NPPA* (*r* = 0.9394, *P* < 0.01), *CACNA2D2* (*r* = 0.9362, *P* < 0.01), *ITGB8* (*r* = 0.9556, *P* < 0.01), and *ITGA5* (*r* = 0.9060, *P* < 0.01) (Table S5).

### Construction of the lncRNA-miRNA-mRNA network

**Fig. 6:**
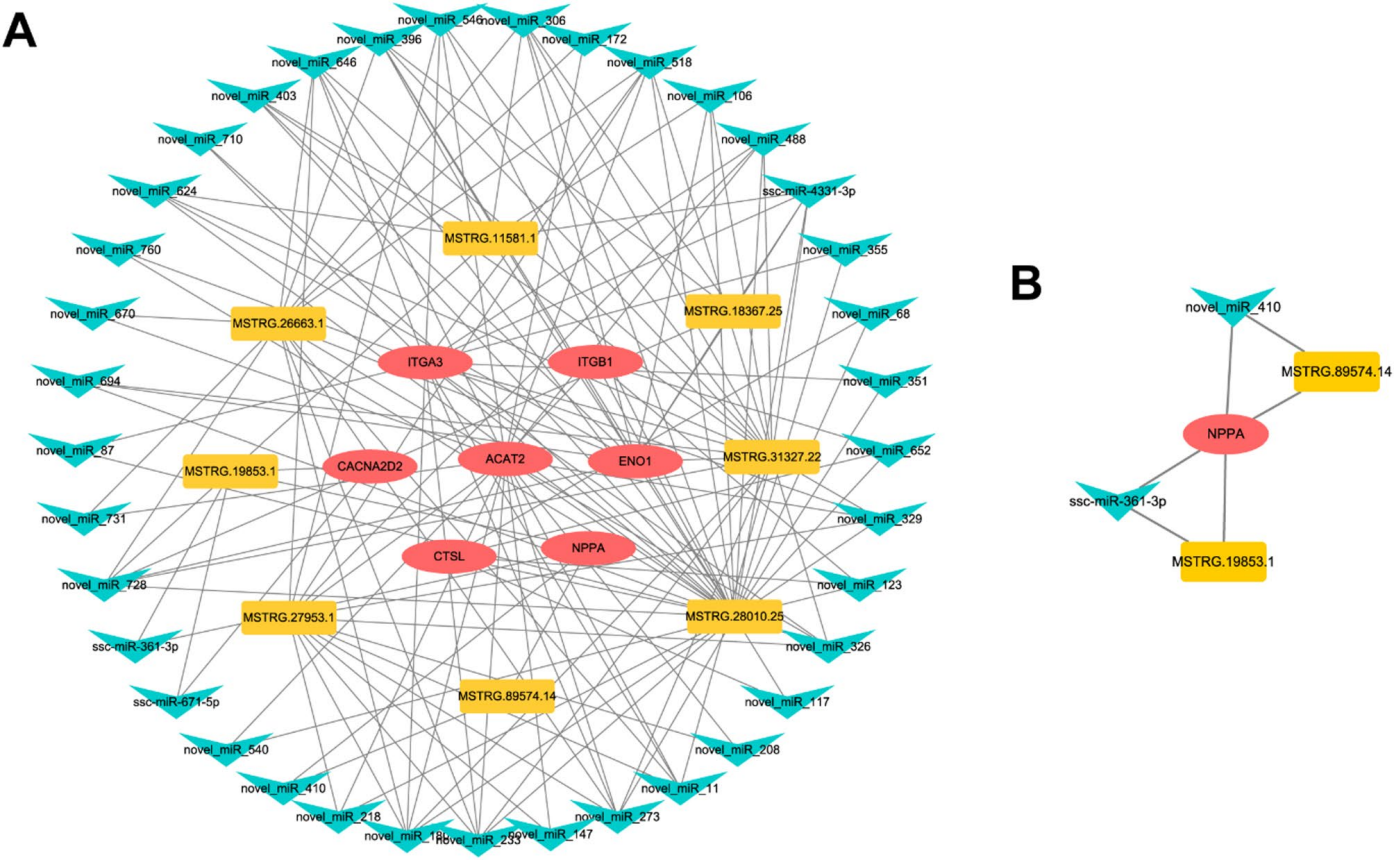
Constructed ceRNA Networks. **A** The lncRNA-miRNA-mRNA ceRNA triple regulatory network is associated with hypoxic adaptation in Tibetan pigs. **B** The ceRNA triple regulatory network interacting with the key gene *NPPA*. lncRNAs, miRNAs, and mRNAs are represented as rectangles, inverted triangles, and ovals, respectively

We constructed a ceRNA network that yielded 97 pairs of candidate lncRNA-miRNA-mRNA interactions from 7 DE mRNAs (*NPPA*, *ENO1*, *ITGB1*, *ITGA3*, *CACNA2D2*, *CTSL*, and *ACAT2*) and 8 DE lncRNAs (MSTRG.19853.1, MSTRG.2663.1, MSTRG.11581.1, MSTRG.18367.25, MSTRG.31327.22, MSTRG.28010.25, and MSTRG.89574.14) through 37 miRNA target-mediated relationships (Fig. 6A, Table S6). It is worth noting that some lncRNAs were targeted by multiple miRNAs. Among these miRNAs, ssc-miR-4331-3p exhibited the highest number of interactions, mediating 6 lncRNA-miRNA-mRNA pairs, compared to an average of 1–5 pairs for other miRNAs. Furthermore, an important hypoxic adaptation-related gene *NPPA*, was found in these regulatory networks. The interactions of the *NPPA* gene with its upstream miRNAs and lncRNAs were captured, and a new regulatory network was constructed. As shown in Fig. 6B, *NPPA* and two lncRNAs (MSTRG.19853.1, MSTRG.89574.14) are regulated by ssc-miR-361-3p and novel-miR-410, respectively.

### Expression levels of DE mRNA, DE lncrna, and DE MiRNA using qRT-PCR

**Fig. 7:**
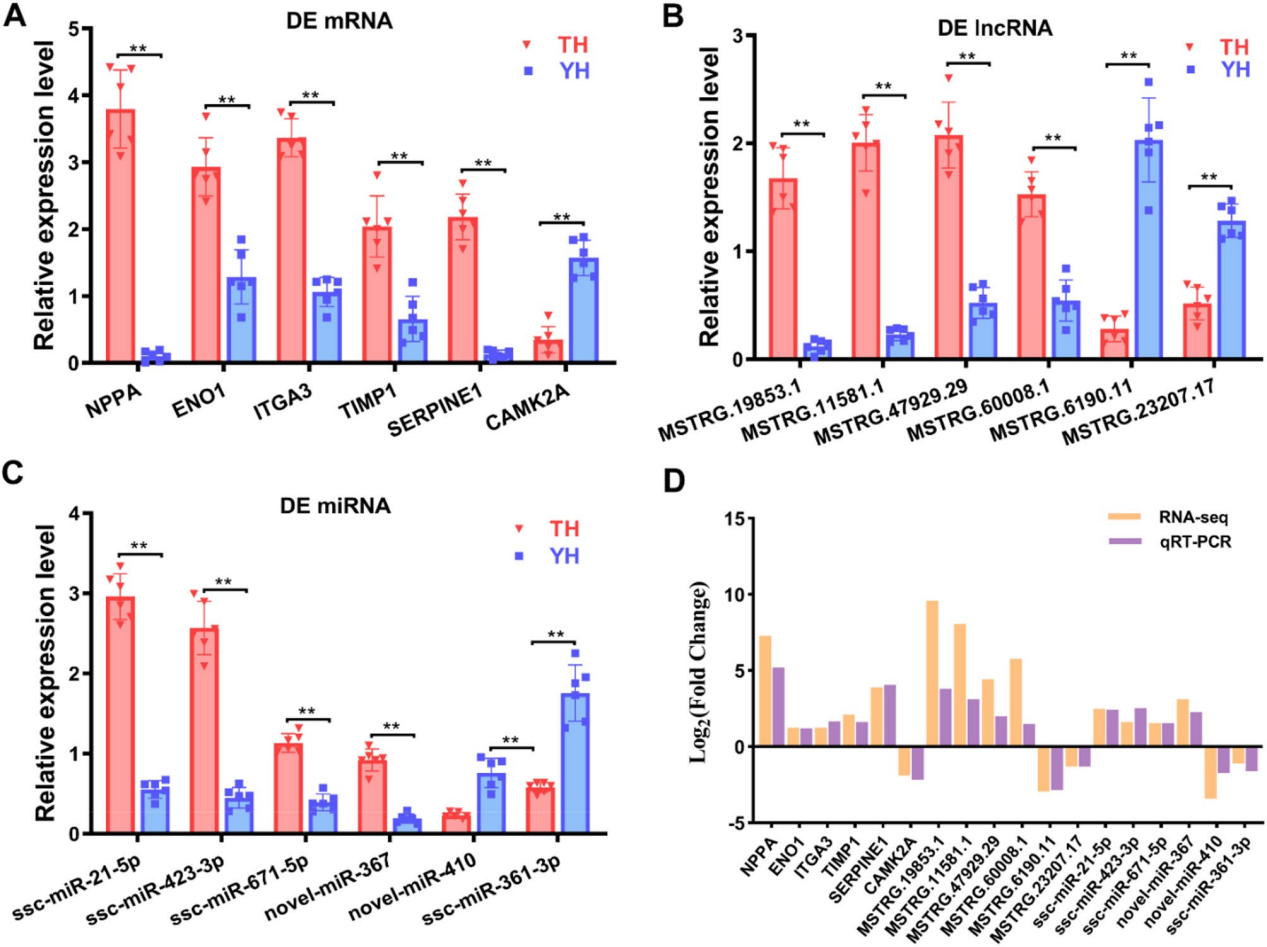
Validating the RNA-seq data by qRT-PCR. **A** The expression of six differentially expressed (DE) mRNAs. **B** The expression of six DE lncRNAs. **C** The expression of six DE miRNAs. Data are represented as the mean ± (standard error) SE. ** on the bars indicate *P* < 0.01 between Tibetan (TH) and Yorkshire (YH) pigs at high-altitude. **D** Comparison of log_2_(fold change) between qRT-PCR and RNA-seq

To validate the DE mRNAs and ncRNAs identified by RNA-seq, six mRNAs (*NPPA*, *ENO1*, *ITGA3*, *TIMP1*, *SERPINE1*, and *CAMK2A*), six lncRNAs (MSTRG.19853.1, MSTRG.11581.1, MSTRG.47929.29, MSTRG.60008.1, MSTRG.6190.11, and MSTRG.23207.17), and six miRNAs (ssc-miR-21-5p, ssc-miR-423-3p, ssc-miR-671-5p novel-miR-367, novel-miR-410, and ssc-miR-361-3p) were selected from the lncRNA-miRNA-mRNA regulatory networks with different expression patterns for qRT-PCR validation (Fig. 7A, B, C). The qRT-PCR expression levels were compared with the expression analysis results of RNA-seq. As shown in Fig. 7D, there was a similarity between the qRT-PCR and RNA-seq analyses regarding the relative expression trends and significance of differential expression for the six mRNAs, six lncRNAs, and six miRNAs. This indicated that transcript identification and abundance estimation were highly reliable.

### The lncRNA-miRNA-mRNA axis was verified by qRT-PCR and dual-luciferase reporter gene system

**Fig. 8:**
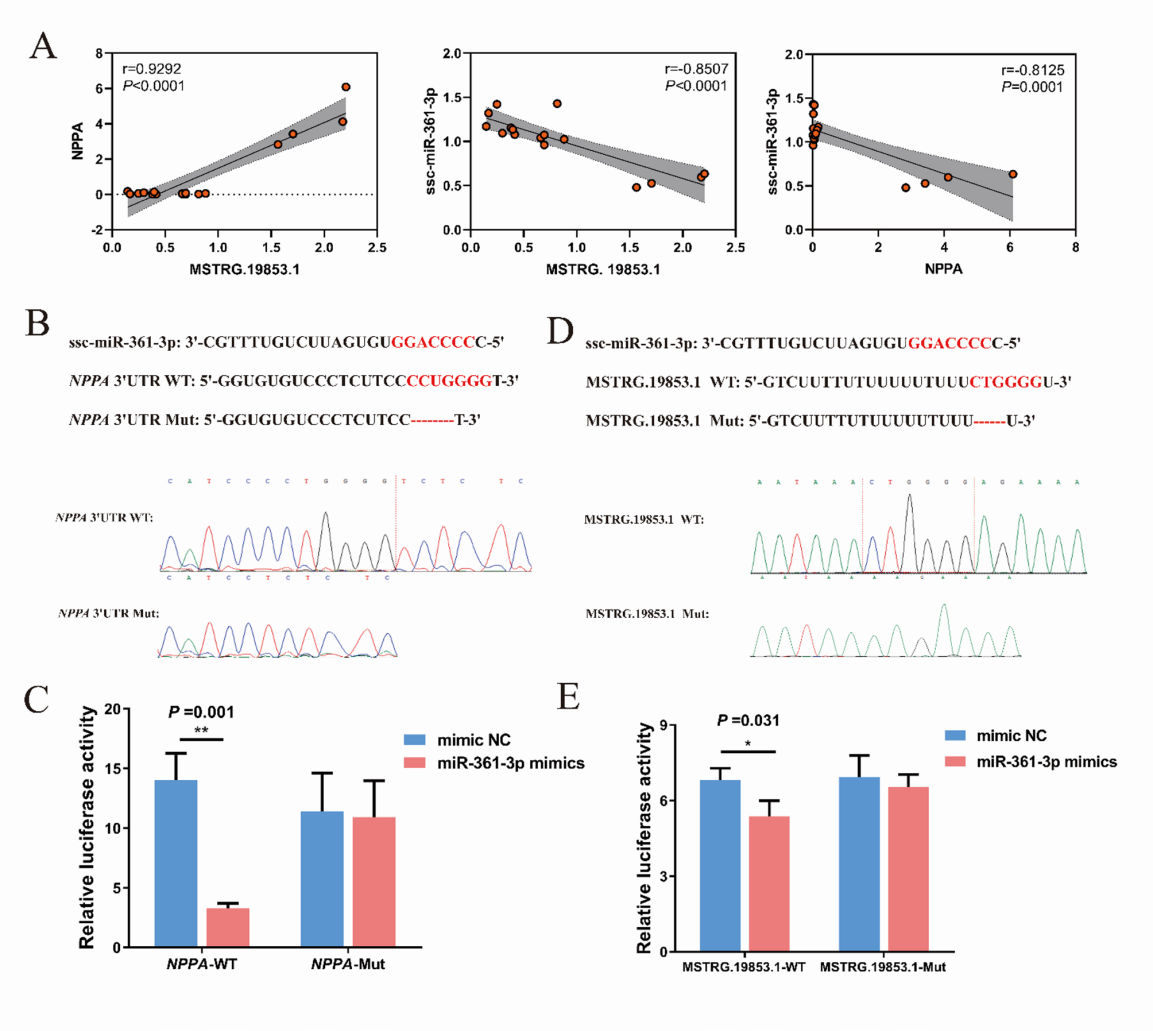
The MSTRG.19853.1/ssc-miR-361-3p/*NPPA* axis was chosen to verify the feasibility of the constructed lncRNA-miRNA-mRNA Network. **A** Correlation analysis of the expression levels of MSTRG.19853.1, ssc-miR-361-3p, and *NPPA* gene from RNA-seq data. The red dots indicate the expression levels of these genes. **B** Potential binding sites of *NPPA* and ssc-miR-316-3p. **C** Luciferase activity assay showed that *NPPA* could bind with miR-316-3p. **D** Potential binding sites of MSTRG.19853.1 and ssc-miR-316-3p. **E** Luciferase activity assay showing that MSTRG.19853.1 could bind with ssc-miR-361-3p. Data are represented as the mean ± (standard error) SE. ** on the bars indicate *P* < 0.01, * on the bars indicate *P* < 0.05

Based on the aforementioned qRT-PCR validation and literature reports, we verified the ceRNA network axis in which *NPPA* was the most interesting gene. MSTRG.19853.1 may interact with ssc-miR-361-3p, and MSTRG.89574.14 may interact with novel-miR-410, both of which target *NPPA*. For these networks, the RNA-seq results showed that *NPPA* was upregulated with a 52.81-fold change, MSTRG.19853.1 with a 91.98-fold change, and MSTRG.89574.14 with a 3.57-fold change in the comparisons of TH vs. YH. qRT-PCR results showed that the expression levels of *NPPA*, MSTRG.19853.1, and MSTRG.89574.14 were significantly higher in the TH group than in the YH group (Fig. 7A and B). In contrast, ssc-miR-361-3p and novel-miR-410 expression levels were lower in the TH group (Fig. 7C). The expression of *NPPA* and MSTRG.19853.1 was significantly positively correlated (*r* = 0.9292, *P* < 0.0001), while MSTRG.19853.1 and ssc-miR-361-3p (*r* = − 0.8507, *P* < 0.0001) and *NPPA* and ssc-miR-361-3p (*r* = − 0.8125, *P* < 0.0001) were significantly negatively correlated (Fig. 8A). However, novel-miR-410 expression levels were decreased in the TH group compared with the YH group (Fig. 7C), which was inconsistent with the trend of MSTRG.89574.14 and *NPPA* expression. The important ceRNA axis MSTRG.19853.1/ssc-miR-361-3p/*NPPA* was identified by further analysis. Bioinformatic prediction tools further demonstrated that ssc-miR-361-3p targeted the 3’UTR of *NPPA* with a complementary binding site (Fig. 8B). Luciferase reporter plasmids containing WT and Mut -*NPPA* were constructed. Co-transfection of the luciferase reporter plasmid containing WT-*NPPA* with ssc-miR-361-3p mimics into DF1 cells resulted in decreased reporter activity (Fig. 8C). Bioinformatic prediction tools also illustrated that ssc-miR-361-3p could target MSTRG.19853.1, with a complementary binding site (Fig. 8D). The luciferase reporter gene assay also validated the molecular interaction between ssc-miR-361-3p and MSTRG.19853.1 (Fig. 8E). Taken together, these results identified the MSTRG.19853.1/ssc-miR-361-3p/*NPPA* axis and demonstrated its role in hypoxic adaptation in Tibetan pigs.

## Discussion

High-altitude environments present intense stresses to living organisms, leading to notable phenotypic and genetic adaptations [45]. Mammals migrating to these regions encounter chronic hypobaric hypoxia, which can result in maladaptive phenotypes such as cardiac hypertrophy and heart failure [23, 46]. Tibetan pigs, having inhabited the Qinghai-Tibetan Plateau for thousands of years, exhibit remarkable genetic adaptations to low oxygen levels, making them an ideal model for studying hypoxic resilience [8]. Recent advancements have highlighted the pivotal role of ncRNAs in regulating hypoxic adaptation and responses [19]. In this study, we aimed to gain insights into the regulatory mechanisms underlying the unique hypoxic adaptation of Tibetan pigs through whole-transcriptome RNA-seq. Additionally, we constructed a hypoxic adaptation-related lncRNA-miRNA-mRNA network hypothesizing that the potential target axis MSTRG.19853.1/ssc-miR-361-3p/*NPPA* serves as a candidate regulatory network for hypoxic adaptation in Tibetan pigs.

The heart, critical for circulatory function, is particularly susceptible to hypoxic stress, which elevates pulmonary vascular resistance and imposes hemodynamic strain [47]. Prolonged hypoxia often triggers pathological right ventricular hypertrophy, as evidenced by increased RVHI in lowland species relocated to high altitudes [48]. This phenomenon is especially pronounced in animals migrating from low to high altitudes. Julian [49] proposed classifications for RVHI: less than 25% was considered normal, 25.0–29.9% was moderate right heart hypertrophy, and greater than 29.9% was severe right heart hypertrophy. Long-term hypoxia is known to induce physiological hypertrophy, an adaptive response that enlarges cardiomyocytes to accommodate increased hemodynamic load, as seen in hypoxia, exercise, or postnatal growth [50–53]. Recent studies have reported increased RVHI, right ventricular fibrosis, and hypertrophy in rats under low-pressure hypoxia [54–58]. In our study, YH exhibited an RVHI > 29.9% and enlarged myocardial fiber size, indicative of maladaptive hypertrophy, whereas TH displayed an RVHI < 25% and normal fiber size, reflecting adaptive cardiac resilience after generations of high-altitude selection. These phenotypic differences, consistent with severe pulmonary hypertension in low-altitude cattle migrated to yak habitats, highlight Tibetan pigs’ robust cardiorespiratory function and their utility as a model for studying hypoxia-adaptive genetic mechanism [59, 60].

To link these physiological observations of cardiac resilience in Tibetan pigs with underlying molecular mechanisms, we explored mRNA and ncRNA expression profiles. Our transcriptomic analysis reveals distinct ncRNA profiles associated with cardiac phenotypes in Tibetan pigs and Yorkshire pigs under high-altitude hypoxia. The lncRNAs identified exhibit fewer exons, lower expression levels, and shorter transcripts and ORF lengths compared to mRNAs, consistent with prior findings [61, 62]. Considering the previously mentioned heart physiological phenotypes, 2,206 DE mRNAs, 795 DE lncRNAs, and 149 DE miRNAs identified in the TH vs. YH comparison group were interesting candidate genes for high-altitude adaptation in Tibetan pigs. This large difference may be attributed to the diverse environmental adaptive selective pressures experienced by Tibetan and Yorkshire pigs during evolution, resulting in inherently greater genetic differences [3, 63]. Our KEGG analysis revealed that DE lncRNA and DE miRNA target genes were significantly enriched in pathways related to hypoxic adaptation, such as DCM, HCM, and HIF-1 signaling. This suggests that lncRNAs and miRNAs may serve as regulatory molecules in heart development during hypoxic adaptation. Furthermore, lncRNAs can regulate gene expression by binding to specific regions of target genes and recruiting transcription factors to activate or repress them [64]. Notably, *NPPA*, enriched in DCM, HCM and HIF-1α pathways, encodes atrial natriuretic peptide, which promotes vasodilation and natriuresis to counteract cardiac hypertrophy [65]. Similarly, *CACNA2D2*, enriched in DCM and oxytocin signaling, regulates calcium signaling in cardiomyocytes, stabilizing contractility under hypoxic stress [66]. These annotations clarify their roles in cardiac adaptation, with *NPPA* promoting vascular remodeling and *CACNA2D2* supporting contractile stability, aligning with TH’s resistance to hypertrophy (RVHI < 25%).

As mentioned earlier, many lncRNAs can function as ceRNAs to sponge miRNAs, further regulating the expression of mRNAs and their related proteins in multiple cellular processes, including hypoxic adaptation at high altitudes [67]. Hypoxia induces significant changes in the expression of hypoxia-related miRNAs by regulating their transcription, biogenesis, and maturation [68]. Several studies have reported miR-361-3p expression in hypoxia-induced heart diseases, indicating that miR-361 may play an essential role in cardiac physiopathology [69, 70]. miR-361-3p is upregulated in resting adult cardiomyocytes during myocardial infarction and promotes cell proliferation [71]. This result suggests that miR-361-3p is an important hypoxic-related miRNA closely associated with ischemic heart disease and may be involved in the hypoxic response of cardiomyocytes. The MSTRG.19853.1/ssc-miR-361-3p/*NPPA* axis represents a pivotal regulatory module in the hypoxic adaptation of Tibetan pigs. MSTRG.19853.1, which is markedly upregulated in TH (log_2_FoldChange = 9.5908), exhibits strong positive correlations with both *NPPA* (*r* = 0.9394, *P* < 0.01) and *CACNA2D2* (*r* = 0.9362, *P* < 0.01). Notably, it may function as a ceRNA by sponging ssc-miR-361-3p (*r* = − 0.8507, *P* < 0.01), thereby alleviating miRNA-mediated repression of *NPPA* (*r* = − 0.8125, *P* < 0.01). *NPPA* is upregulated in TH, consistent with its protective role in high-altitude adaptation observed in Ethiopian highlanders, where it supports cardiac contraction and vascular integrity under hypoxia [72]. In contrast, low *NPPA* expression in YH may contribute to maladaptive hypertrophy, highlighting its anti-hypertrophic function [9]. Dual-luciferase reporter assays conducted in DF1 cells provided preliminary validation of this regulatory axis. However, future studies employing primary porcine cardiomyocytes would significantly enhance the physiological relevance of these findings. Given that cis-regulatory mechanisms are effective in only approximately 50–65% of cases, computational predictions may include false positives [73]. Therefore, additional experimental validations using CRISPR interference, RNA pull-down, and RNA immunoprecipitation assays are warranted to confirm direct interactions within this ceRNA network [74].

High-altitude species, including Tibetan pigs, yaks, Tibetan chickens, and Tibetan sheep, have evolved sophisticated molecular adaptations to chronic hypoxia, with ncRNAs and hypoxia-inducible factor pathways playing conserved roles across species [75]. In Tibetan pigs, our study identifies the MSTRG.19853.1/ssc-miR-361-3p/*NPPA* axis as a key regulator of cardiac protection, potentially the first to link *NPPA* to porcine hypertrophy resistance. Similarly, yaks employ lncRNAs to modulate pulmonary vascular remodeling and HIF-1α-mediated angiogenesis [19, 76], while Tibetan chickens utilize ncRNA-driven angiogenesis and blood circulation [67, 77]. Tibetan sheep also exhibit ncRNA-mediated oxidative stress responses [78]. These adaptations, while organ-specific in emphasis—cardiac in pigs, pulmonary in yaks, circulatory in chickens, and systemic in sheep—converge on shared molecular pathways. The Tibetan pig ceRNA network integrates *NPPA* and other hypoxia-related genes with KEGG-enriched processes like DCM, HCM, and HIF-1 signaling, indicating that MSTRG.19853.1 may regulate cardiac remodeling and hypoxic adaptation through these pathways. Comparable ncRNA-HIF-1α crosstalk in yaks, chickens, and sheep underscores a conserved molecular framework for hypoxic resilience [79, 80]. This cross-species similarity highlights that, despite phenotypic differences, high-altitude adaptations rely on homologous mechanisms, including ncRNA-mediated redox homeostasis and vascular remodeling. These findings provide a balanced perspective on ncRNA-driven adaptation in plateau mammals, moving beyond single-organ studies to reveal shared genetic strategies [81].

This study has limitations that merit consideration. The use of three biological replicates per group, constrained by high-altitude sampling logistics, may elevate false-positive risks for low-abundance ncRNAs. Stringent statistical thresholds mitigate this, but larger cohorts are needed for validation. The absence of low altitude controls limits our ability to attribute transcriptomic differences solely to hypoxia, as species-specific genetic variation may contribute [82]. While TH’s long-term adaptation and YH’ s maladaptive hypertrophy (RVHI > 29.9%) strongly implies hypoxia-driven effects, future studies comparing both species at low altitude and high altitude will clarify these contributions. The heart-focused analysis captures a critical hypoxia-responsive organ but overlooks heart-lung crosstalk, which may amplify systemic adaptation [83]. Preliminary evidence suggests *NPPA* influences pulmonary vascular remodeling, prompting planned studies to examine the MSTRG.19853.1/ssc-miR-361-3p/*NPPA* axis in pulmonary tissues. Our findings illuminate the genetic underpinnings of hypoxic adaptation in Tibetan pigs, offering a comparative framework for studying plateau mammals. The MSTRG.19853.1/ssc-miR-361-3p/*NPPA* axis represents a candidate mechanism for cardiac resilience, with parallels in other high-altitude species. Future studies will investigate whether modulating this axis enhances hypoxia tolerance in non-adapted mammals, potentially informing comparative studies for high-altitude physiology. By bridging ncRNA dynamics with cardiac phenotypes, this study advances our understanding of hypoxic resilience in plateau animals, with implications for evolutionary biology and comparative genomics.

## Conclusions

This study unveils a hypoxia-responsive ceRNA network in Tibetan pigs, with the MSTRG.19853.1/ssc-miR-361-3p/*NPPA* axis orchestrating cardiac adaptation to high-altitude hypoxia. Through *NPPA* upregulation and ssc-miR-361-3p suppression, Tibetan pigs resist pathological hypertrophy, contrasting with Yorkshire pigs’ maladaptive response. These insights enhance our understanding of ncRNA-mediated hypoxic adaptation in plateau mammals and position *NPPA* as a candidate for further study in high-altitude physiology, pending in vivo validation. Future multi-organ and larger-scale studies will refine these findings, advancing comparative research on hypoxic resilience.

## Supplementary Information

Supplementary Material 1: Table S1. Primers used for qRT-PCR and dual-luciferase reporter gene system validation.

Supplementary Material 2: Table S2. Summary of sequencing reads aligned with the Sus scrofa genome.

Supplementary Material 3: Table S3. The 2,206 differentially expressed mRNAs (DE mRNAs), 795 DE lncRNAs, and 149 DEmiRNAs.

Supplementary Material 4: Table S4. GO and KEGG enrichment of differentially expressed lncRNA (DE lncRNA) and DE miRNA target genes.

Supplementary Material 5: Table S5. Key DE-lncRNA/DE-mRNA pairs.

Supplementary Material 6: Table S6. Potential key DE-mRNA, their targeted DE-miRNA/DE-lncRNA related to hypoxic adaption in the Tibetan pigs.

## Data Availability

The raw data of RNA sequencing is available at the NCBI GenBank Sequence Read Archive (SRA), under accession number PRJNA880668.

## Abbreviations

ncRNAs: Non-coding RNAs
DE lncRNAs: Differentially Expressed lncRNAs
DE mRNAs: Differentially Expressed mRNAs
DE miRNAs: Differentially Expressed miRNAs
TH: Tibetan pig living at High-altitude
YH: Yorkshire pig living at High-altitude
ceRNA: Competitive endogenous RNA
qRT-PCR: Quantitative real-time PCR
WV: Whole Ventricle
RV: Right Ventricle
RVHI: Right Ventricular Hypertrophy Index
H&E: Hematoxylin and Eosin
rRNA: Ribosomal RNA
FPKM: Fragments Per Kilobase of exon model per Million mapped fragments
CPC2: Coding Potential Calculator
CNCI: Coding-Non-Coding Index
FDR: False Discovery Rate
tRNA: Transfer RNA
snoRNA: Small nucleolar RNA
snRNA: Small nuclear RNA
TPM: Transcripts Per kilobase of exon model per Million mapped reads
ORF: Open Reading Frame
KEGG: Kyoto Encyclopedia of Genes and Genomes
PCA: Principal Component Analysis
DCM: Dilated Cardiomyopathy
HCM: Hypertrophic Cardiomyopathy
ARVC: Arrhythmogenic Right Ventricular Cardiomyopathy
